# Broadband Mueller ellipsometer as an all-in-one tool for spectral and temporal analysis of mutarotation kinetics

**DOI:** 10.1039/d3ra00101f

**Published:** 2023-02-27

**Authors:** Daniel Vala, Martin Mičica, Daniel Cvejn, Kamil Postava

**Affiliations:** a IT4Innovations, National Supercomputing Center, VSB – Technical University of Ostrava 17. listopadu 2172/15 708 00 Ostrava-Poruba Czech Republic daniel.vala@vsb.cz kamil.postava@vsb.cz; b Faculty of Materials Science and Technology, VSB – Technical University of Ostrava 17. listopadu 2172/15 708 00 Ostrava-Poruba Czech Republic; c Laboratoire de Physique de l’École Normale Supérieure, CNRS UMR 8023 24 rue Lhomond 75005 Paris France; d ENET Centre, CEET, VSB – Technical University of Ostrava 17. listopadu 2172/15 708 00 Ostrava-Poruba Czech Republic

## Abstract

Spectroscopic Mueller matrix ellipsometry is becoming increasingly routine across physical branches of science, even outside optics. The highly sensitive tracking of the polarization-related physical properties offers a reliable and non-destructive analysis of virtually any sample at hand. If coupled with a physical model, it is impeccable in performance and irreplaceable in versatility. Nonetheless, this method is rarely adopted interdisciplinarily, and when it is, it often plays a supporting role, which does not take benefit of its full potential. To bridge this gap, we present Mueller matrix ellipsometry in the context of chiroptical spectroscopy. In this work, we utilize a commercial broadband Mueller ellipsometer to analyze the optical activity of a saccharides solution. We verify the correctness of the method in the first place by studying the well-known rotatory power of glucose, fructose, and sucrose. By employing a physically meaningful dispersion model, we obtain 2π-unwrapped absolute specific rotations. Besides that, we demonstrate the capability of tracing the glucose mutarotation kinetics from just one set of measurements. Coupling the Mueller matrix ellipsometry with the proposed dispersion model ultimately leads to the precisely determined mutarotation rate constants and spectrally and temporally resolved gyration tensor of individual glucose anomers. In this view, Mueller matrix ellipsometry may stand as an offbeat yet equal technique to those considered classical chiroptical spectroscopy techniques, which may help open new opportunities for broader polarimetric applications in biomedicine and chemistry.

## Abstract 2

We utilize a commercial broadband Mueller ellipsometer to monitor the kinetics of glucose mutarotation through the dispersion of its optical activity.

## Graphical abstract

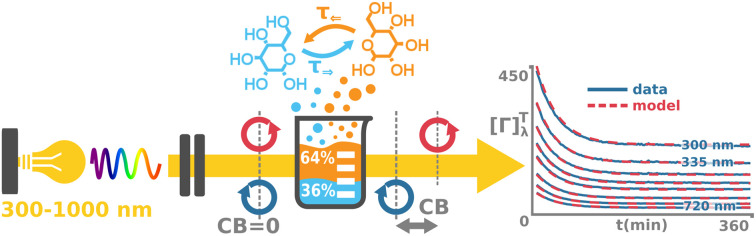

## 1 Introduction

In recent years, cutting-edge applications of Mueller matrix spectroscopic ellipsometry (MMSE) have been bursting far beyond its homeland of photonics and material sciences, bringing us probes into cancer diagnostics,^1–3^ ultrafast electronic processes,^4,5^*in vivo* imaging,^6,7^ solar and hydrogen energy harvesting,^8–10^ toxin detection,^11^ and much more. The unique flexibility of MMSE stems from the contactless ability of light polarization to track even the subtlest material attributes. It is virtually the signature of the interplay between circular polarizations and chiral objects, providing us with experimental observables and elucidating their spatial conformation at the molecular level. Specifically, different travel speeds of left- and right-circular polarizations in a given medium reflect its optical activity (OA). In the light of the MMSE, we directly observe a Mueller matrix – a characteristic quantity embracing OA dispersion (optical rotation) and its Kramers–Kronig related absorptive counterpart (circular dichroism).

The leading chiroptical techniques are mainly vibrational circular dichroism^12,13^ (VCD), Raman optical activity^14–16^ (ROA), and electronic circular dichroism^17^ (ECD), which, in conjunction with *ab initio* calculations,^18^ reveal the absolute configuration of a molecule. Within those, it seems quite ambitious to justly pick MMSE and challenge the well-established methods. However, contemporary ellipsometers have come a long way from the traditional single-wavelength polarimetry (saccharimetry), particularly in greater spectral range, high accuracy, capability of real-time tracking of the optical response, efficient data processing, and much more. Bottom-line, putting the polarimetric techniques into the context of state-of-the-art MMSE would open up new opportunities in chiroptical spectroscopy.

Covering the frequency range typically from ultraviolet to the infrared spectrum, the Mueller matrix reflects both electronic and vibrational transitions responsible for the chiroptical response of a chiral molecule. As a rule of thumb, the ellipsometric community has adopted the glucose molecule as a universal representation of a chiral object within the landscape of chemistry. Its clinical implications have attracted attention exploiting MMSE versatility by sensing glucose^19^ in media simulating scattering events,^20–22^ body-fluid environments,^23^ or even phantom tissues^24^ and actual human tissues.^25^ Moreover, fast and precise response makes MMSE widely popular as an *in situ* control^26,27^ capable of real-time tracking of dynamic processes. This has been done very sparsely in chemical kinetics,^28^ and only once regarding glucose mutarotation,^29^ despite being fundamental to saccharides. Considering that the used method is monochromatic and does not account for glucose gyration spectra, it leaves much room for improvement, where MMSE appears to be a hot candidate.

In this article, we present commercially available MMSE as an up-to-date tool for non-destructive sensing of saccharide chiroptical response capable of tracking the mutarotation kinetics. First, we will show the power of the spectral Mueller matrix in tandem with a physically consistent model. The correct fit to the experimental data enables retrieval of the isotropic gyration tensor components spectra, gaining the sensitivity to 2π-unwrapped absolute optical rotation. A simple combination of the spectral approach with the rate equation promotes the proposed method to the next level. We will show that the MMSE tracing of just a single forward mutarotation reaction is sufficient enough to reveal (i) absolute optical rotations at all measured wavelengths at any time instant, (ii) spectrally and temporally resolved effective gyration response of the measured solution, (iii) mutarotation rate constants and corresponding concentrations evolution, and, remarkably, (iv) gyration spectra of individual α- and β-anomers of d-glucose. These demonstrate the paramount strength of the proposed method as a chiroptical technique for quick, easy-to-use, and reproducible analysis of various static and dynamic processes in physical chemistry, potentially providing an experimental counterpart to specific quantum-mechanical quantities employed in *ab initio* modeling of OA.^30^

Note, right before we focus on the main results (Section 3), the next section introduces the necessary technicalities of the method.

## 2 Methods

### 2.1 Sample preparation

d-(+)-Glucose (or d-glucose, anhydrous, ≥99% purity), d-(−)-fructose (or d-fructose, anhydrous, ≥99% purity), and d-(+)-sucrose (or d-sucrose, anhydrous, ≥99% purity) from the Lach-Ner Group, and α-d-(+)-glucose (or α-d-glucose, anhydrous, 96%) from Sigma-Aldrich were used in this study without further purification. Saccharide powders were dissolved and thoroughly mixed in an appropriate amount of distilled water in volumetric flasks to yield concentrations *C* of 0.25 mol dm^−3^ ≡ 0.25 M, 0.50 M, and 3.00 M.

Before measuring the spectral responses of d-glucose and d-fructose, their solutions were first left at rest for 24 hours to reach the state of their anomeric equilibrium at a given temperature. Solutions of d-sucrose, which is a non-reducing and therefore mutarotation inactive saccharide, were measured just after 4 hours, only to ensure perfect dissolution. Mutarotation ellipsometric experiments on α-d-glucose were performed right away with freshly prepared solution.

### 2.2 Mueller matrix ellipsometry

The method principle reflects its very name – the measurement of the change in the arbitrary (generally elliptical) polarization state of light after interaction with a sample. The polarization state change records a 4 × 4 Mueller matrix **M**, which reflects the complete polarimetric response of a sample, including the effect of optical activity in chiral liquids.

**Fig. 1:**
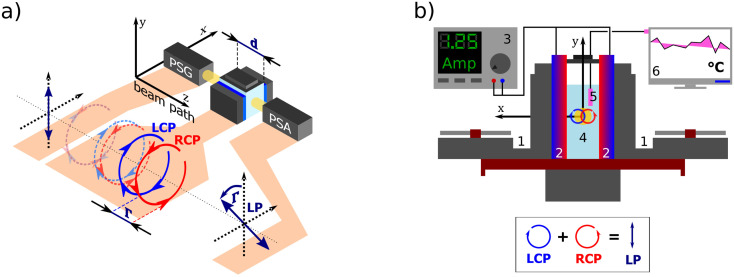
(a) A polarization state generator (PSG) emits polarized light including circular. If propagating through an optically active solution, the left and right circularly polarized eigenstates phase-shift (we say they are circularly retarded) by the amount equal to *Γ*. This retardation manifests itself as a rotation of the plane of the linearly polarized light by *Γ*, see Appendix A.2, which is detected in the polarization state analyzer (PSA). (b) Scheme of the custom-developed sample stage. A pair of cooled (1) Peltier devices (2) with adjustable input current (3), which provide temperature stability inside the cuvette with the measured solution (4). The temperature is sensed by a PT100 sensor (5) and recorded in real-time (6).

The corresponding reduced (normalized to *m*_11_) Mueller matrix **M**1
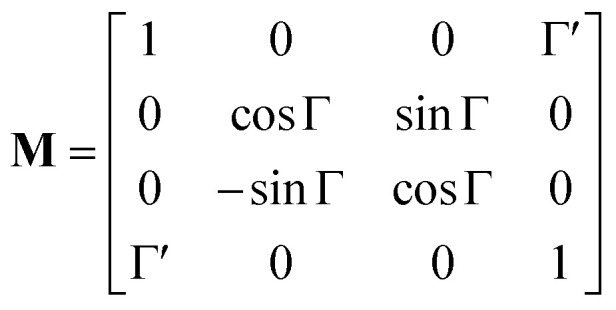
accounts for the influence of OA in the form of its dispersive part *Γ*(*λ*) and absorptive part *Γ* ′(*λ*). The OA spectral (wavelength, *λ*) dependence is connected with the different propagation speeds of left (L) and right (R) circularly polarized light, or eigenmodes (those states of a polarized light that do not change their polarization state when passing through a given environment), in a medium of length *d*. It is caused by different dispersion of L and R eigenmodes refractive indices *n*_L,R_. The delay between the eigenmodes, called circular phase retardation, effectively manifests itself as a rotation of the plane of linearly polarized light (see Fig. 1a) over the angle2
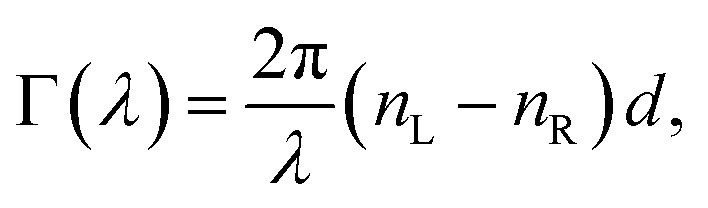
which corresponds to the traditional definition of OA. Refer to Appendix A.1 for details. Of course, this definition is consistent with the theory on the microscopic optical activity approach accounting for chiral molecules with random orientation dissolved in an achiral solution, refer to Appendix A.2 for details.

All experimental work was done on a standard Woollam RC2-DI (dual-rotating compensator) Mueller matrix ellipsometer in transmission configuration with the spectral range of 193 nm–1700 nm (6.42 eV–0.73 eV). Its instrumentation is based on a polarization state generator (PSG – probing arm: source, polarizer, and rotating compensator) and polarization state analyzer (PSA – detection arm: rotating compensator, analyzer, detector part).

### 2.3 Temperature cell design and control

For temperature-controlled measurements, we have designed a custom Peltier device-based 3D-printed cuvette holder. Fig. 1b shows the cell schematics. The keystone element of the temperature control is a pair of Peltier modules (HP-127100HTS, 90 W, temperature range from −20 °C to +200 °C) with each module attached to one side of the optical glass cuvette with the optical path length 50.04 mm working as a heat pump. The Peltier modules are powered by a current source (BOP20-10M Kepco Bipolar Power Supply) in a parallel circuit. The actual temperature of the measured solution is sensed in real-time by a PT100 temperature sensor connected to a PT-104 data logger.

We connected the outer sides of the Peltier modules to a pair of Corsair H45 computer water cooling units to ensure their stable temperature. The second critical aspect of our setup is a good thermal conduction between each contact of the Cuvette–Peltier–Cooler unit. To do so, we encased all these parts in a 3D-printed exoskeleton allowing us to bolt them together and apply a thermal paste. The system is equipped with a small magnet attached to an electric motor under the cuvette, which can be used as a magnetic stirrer to ensure a homogeneous temperature distribution in the solution.

### 2.4 Mutarotation experiment methodology and rate equation

Mutarotation is a change in the concentrations of the α-anomer and β-anomer of reducing saccharides upon dissolution in water. The presence of the hemiacetal carbon causes, through ring-chain tautomerism, a gradual inter-conversion between the α- and β-anomers until equilibrium is established. It results in a change in the optical rotation of a given solution.

Fig. 3a shows the schematics of the glucose mutarotation. It is not often pointed out that a tiny fraction of the six-membered rings (pyranoses) convert to five-membered rings (furanoses) during mutarotation, regardless of the starting substance from which the solution is prepared. The system at equilibrium remains a dynamic environment in which inter-conversions are constantly occurring over and over, yet the overall concentration ratio among all forms is conserved. The first effect mentioned is negligible as far as the concentrations of the five-membered rings are concerned, and the second doesn’t affect the experiment. Therefore, we do not include either effect in the description of mutarotation.

The mutarotation of d-glucose is described by the reverse reaction3
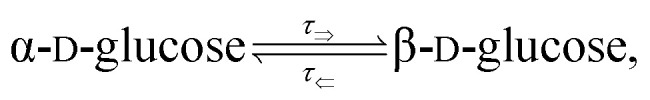
where *τ*_⇒_ and *τ*_⇐_ are forward and reverse mutarotation rate constants, respectively. The reaction kinetics evaluates the change in α- and β-anomer concentrations *C*_α,β_ and is given by a first-order rate equation4
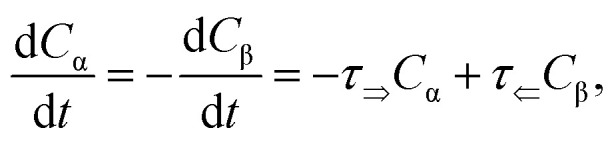
with initial conditions

5a *C*_β_(*t* = 0) = 0,

5b *C*_α_(*t* = 0) = *C*,

5c *C*_α_(*t*) + *C*_β_(*t*) = *C*,

and solution

6 *C*_α_(*t*) = *C*_α,eq_ + (*C* − *C*_α,eq_) exp (−*τt*),

where *C*_α,eq_ is the α-anomer concentration in equilibrium. The observed total rate constant

7 *τ* ≡ *τ*_⇒_ + *τ*_⇐_

characterizes the time evolution of d-glucose α- and β-forms instantaneous concentrations *C*_α,β_*via* forward and reverse rate constants *τ*_⇒_, *τ*_⇐_, respectively, and equilibrium constant *K*,8a
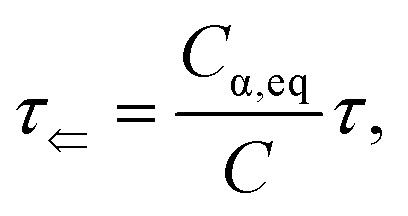
8b
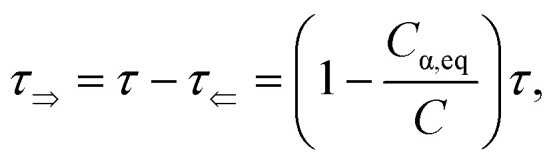
8c
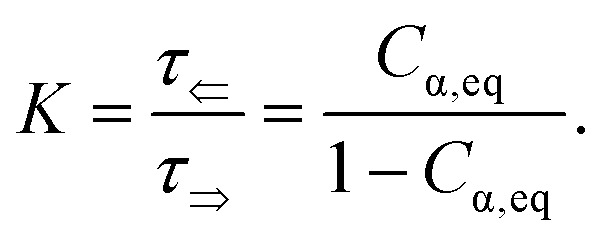


We set the initial time *t* = 0 of the α-d-glucose mutarotation process to the instant at which crystals were no longer visible in the aqueous solution. It takes approximately 30 s for concentrations of *C*_α_ = *C* = 0.25 M. The first ellipsometric measurement was taken after 300 s. We monitored the temperature *T* inside the cuvette throughout the process with the accuracy of 0.5 °C for the following 12 hours, at which point the experiment was terminated.

## 3 Results

### 3.1 Spectral model and method validity

**Fig. 2:**
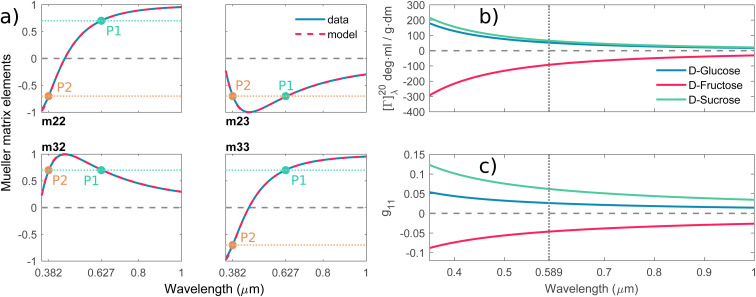
(a) Spectra of the Mueller matrix key elements (*m*_22_, *m*_23_, *m*_32_, *m*_33_), corresponding to the measurement of a 3 M d-fructose solution. The experimental data (blue curve) are compared with the spectral model (red curve) and are in good agreement. Points P1 and P2 correspond to different values of specific rotatory power, which we can accurately determine. (b) Calculated values of specific rotatory powers for d-glucose (blue curve), d-fructose (red curve) and d-sucrose (green curve). We demonstrate the quality of the method and model on well-known values for a wavelength of 589 nm, see Table 1. (c) Dispersion of the gyration tensor element *g*_11_ for saccharides respective to Fig. 2b.

Fig. 2a shows typical spectra of elements *m*_22_, *m*_23_, *m*_32_, *m*_33_ of the Mueller matrix (1) corresponding to measurement of an optically active solution, in this particular case, the 3 M d-fructose solution. The experimental data (blue curves) are in agreement with the model (red curves). Eqn (2) does not constitute an optimal model for subsequent minimization against the experimental data, since the difference *n*_L_ − *n*_R_ incorporates the effective cumulative response of both medium refractive index dispersion *n* and chirality dispersion.

**Fig. 3:**
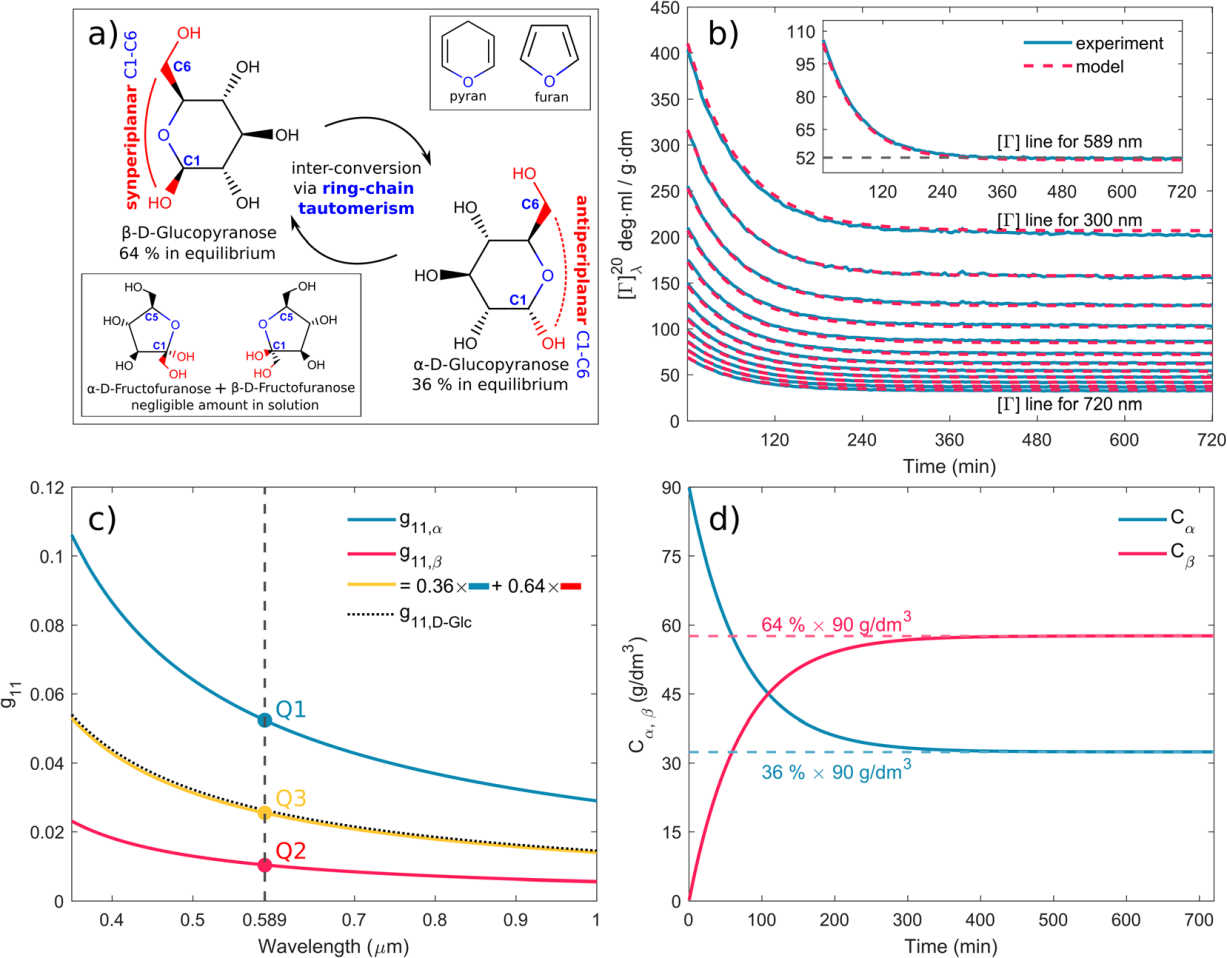
(a) Schematic of d-glucose mutarotation. We consider six-membered pyranose rings only, as the five-membered furanose forms can be neglected. (b) Time evolution of [*Γ*]^20^_*λ*_ curves for selected wavelengths. The experimental data are compared to a phenomenological dispersion model (11), allowing determination of the value of the specific rotation at any time instant of mutarotation for any wavelength in the wavelength range of 300–1000 nm. The inset figure shows [*Γ*]^20^_589_(*t*) converging to a value of ≈52 ml g^−1^ dm^−1^. (c) Fitted dispersions of the gyration tensor elements separately for α- and β-d-glucose. Their weighted summation (yellow curve) fits well with the effective gyration of d-glucose (dotted curve). (d) Time evolution of α- and β-d-glucose concentrations in water solution.

Therefore, we propose to apply the dispersion model that combines an optics-based approach based on oscillator models,^34,35^ accounts for material symmetry defining the shape of material (permittivity, gyration) tensors,^36,37^ and employs rigorous wave-equation-based calculations of the polarization eigenmodes,^38,39^9a
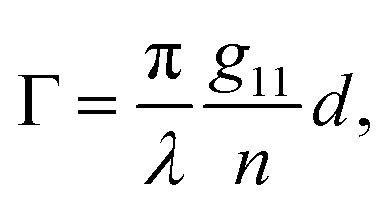
9b
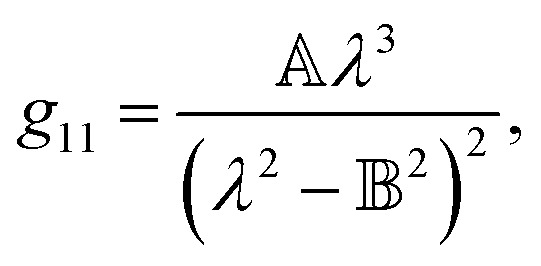
9c
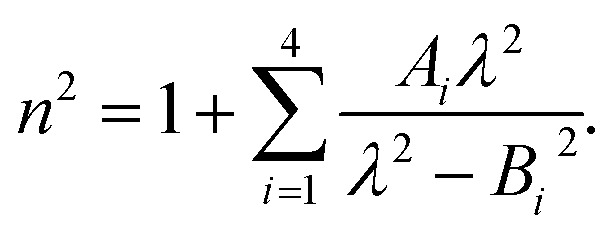
The model separates the gyration tensor element *g*_11_ from the medium refraction index *n*, both described by phenomenological constants 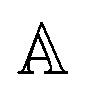, 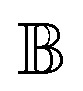, *A*_*i*_, *B*_*i*_, of which the first two had set as the fitting parameters. The constants *A*_*i*_, *B*_*i*_ describing *n* of water were taken from ref. 40. Note that the refractive index of water differs from that of a mixture of water and saccharide, however, the difference is negligible for dilute solutions^41^ – 5% change in saccharide to water mass ratio causes less than 1% change in the refractive index dispersion (the mass ratio of 0.25 M saccharide solution is approximately 4.5%). The spectral limitation of the measurement comes from the transparency of the optical glass cuvette (>300 nm) and the water absorption (>1000 nm). Since monosaccharides exhibit electronic circular dichroism in the spectral region below 190 nm, this is a phenomenon outside the spectral range of the ellipsometer employed. Thus, *Γ* ′(*λ*) = 0.

The striking advantage of having spectral measurements is the sensitivity to the absolute (2*k*π-wise, 
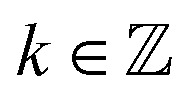
 ) optical rotation of studied media. It gives the flexibility to measure very high concentrations of the solution (several mol l^−1^), compounds with enormous optical activity (*e.g.*, helicenes), or both. Fig. 2a shows a practical example. In the spectra of **M** of the 3 M solution of d-fructose, we have marked points P1 and P2 corresponding to measurements at randomly picked wavelengths of 382 nm, and 627 nm. The first advantageous feature of the spectral Mueller matrix **M** is the complementarity between the elements *m*_22_, *m*_33_ and *m*_23_, *m*_32_ resulting from the evenness and oddness of the sine and cosine functions. Thus, based on the combination of the signs of the analyzed elements of **M**, it is possible to unambiguously determine *Γ*(P1, P2) corresponding to the spectral points P1 and P2 in Fig. 2a, yet only within the 2π range. The second aspect is the dispersion model. Its parameters are specific for every material, varying in the amplitude, curvature, and offset of the observed dispersion, and their combination is unique to each point in the spectrum. These properties allow us to unmistakably distinguish – in absolute values without any arbitrariness of the 2*k*π shift – the values of *Γ* at all measured points in a wavelength range with any dense spectral oscillations. In this case, *Γ*(P1) = −0.38 rad = −22.62°, and *Γ*(P2) = −0.38 rad − π/4 = −67.25°. Apparently, monochromatic methods or methods with a limited spectral range do not allow this. The sign of the observed rotation reflects the handedness of the saccharides.

In addition, the dispersion model enables us to distinguish the data from noise or systematic measurement errors even for particularly weak signals. Considering that the Woollam RC2 theoretical threshold value for distinguishing signal from noise in the Mueller matrix is 0.001, the lowest detectable *Γ* = 0.5 mdeg. This value corresponds to the lowest measurable concentrations of d-glucose (19.0 mg ml^−1^), d-fructose (10.8 mg ml^−1^) and d-sucrose (15.3 mg ml^−1^) at 589 nm.

Fig. 2b shows d-glucose, d-fructose and d-sucrose optical rotatory power spectra [*Γ*]^*T*^_*λ*_ defined as10
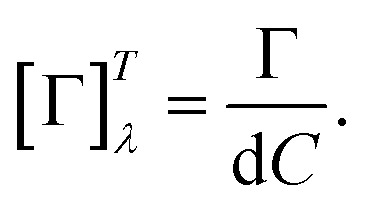
We have particularly indicated [*Γ*]^20^_589_ (measured at the temperature *T* = 20 °C and *λ* = 589 nm), as these are generally well-known values of specific rotatory power obtained by monochromatic setups working with a sodium D-line lamp. A comparison of the measured values with those reported in the literature is provided in Table 1 and shows quite a good agreement between them. The deviations could be mainly due to minor errors caused by weighing and solution preparation. Fig. 2c shows the fitted *g*_11_ dispersions of the studied saccharides.

**Table 1:** Selected specific rotation values [*Γ*]^20^_589_ of studied saccharides. The values are in (deg ml g^−1^ dm^−1^)

Saccharide	This work	Ref. 31	Ref. 32	Ref. 33
d-Glucose	52.68	52.7	52.7	52.3
d-Fructose	−92.53	−92.0	−92.0	−93.0
d-Sucrose	65.36	66.37	65.9 to 66.49	66.5

Up until now, we have been considering the effective cumulative optical response and describing the studied materials accordingly. In the above-studied cases, the physical meaning of corresponding dispersions *g*_11_ (see Fig. 2c) is not directly the response of pure substances (pure anomers) but rather an effective value for a given solution in equilibrium, which may consist of an arbitrarily high number of chiral chemical individuals. Given this logic, each individual in the solution manifests its own *g*_11_. This approach is crucial for the study of mutarotation, as it gives us access to all anomers involved.

### 3.2 Application of the ellipsometry to mutarotation kinetics

We assume that the observed gyration response of the solution is proportional to the concentration-weighted gyration response of the individual anomers in the solution:11
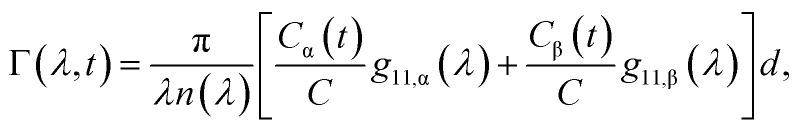
where *g*_11,α_ and *g*_11,β_ are gyration tensor dispersions of pure α-anomer and β-anomer of d-glucose. Such a combination of the spectral model (9) and the rate equation (6) allows for a physical description of *Γ*(*λ*,*t*) based on (2).

#### 3.2.1 Spectral dependence

Fig. 3b shows model (eqn (11), red curves) optimized to the experimental data (blue curves) of the α-d-glucose mutarotation process. Each curve stands for the [*Γ*]^20^_*λ*_ time evolution of 0.25 M α-d-glucose solution during ongoing mutarotation. For better clarity, we show results for selected wavelengths only – the actual measured and modeled spectral range is 300–1000 nm with the step of 1 nm. As expected, the observed spectra follow the exponential tendency given by (6) and converge to the value of [*Γ*]^20^_*λ*_(*t* → ∞) corresponding to the equilibrium state between the anomers in the solution. The inset of Fig. 3b shows the [*Γ*]^20^_589_(*t*) curve converging to the value [*Γ*]^20^_589_(*t* = 720 min) ≈ 52 deg ml g^−1^ dm^−1^, which is consistent with those in Table 1.

Now, with the modeled spectra available, we dig more into their physical significance. We present the individual gyration tensor contributions *g*_11,α_(*λ*) and *g*_11,β_(*λ*), see Fig. 3c. In the following, we will address the validity of our findings: the ellipsometric measurements start with a freshly prepared solution of α-d-glucose and the measured data initially contains mainly information about the dispersion of *g*_11,α_, which we are thus able to model accurately. As the mutarotation reaction progresses, the effect of *g*_11,β_ dispersion gets more dominant in the optical response of the system ever closer to its equilibrium state. The consequence is that the *g*_11,β_ is not accessible in a portion of measurements causing lower numerical sensitivity to this contribution, which accounts for the correlation between the parameters describing *g*_11,β_ and *C*_α,eq_. In order to correctly determine the dispersion of *g*_11,β_ at all, we have to assume the known value of *C*_α,eq_, in this case *C*_eq,α_ : *C*_eq,β_ = 36% : 64% at *T* = 20 °C. Elsewise, the dispersions of *g*_11,β_ and *C*_α,eq_ correlate in the model. This step bears the risk of possible inaccuracies and should be justified: applying the discussed ratio, the gyration response of the equilibrium solution is equal to the concentration-weighted sum of the gyration spectra of the isolated anomers (blue and red curves in Fig. 3c) resulting in the yellow curve. In other words, this curve must reproduce (and it actually does with 97.5–99% accuracy) the gyration response of previously measured d-glucose (black dotted curve) as in Fig. 2c. Nonetheless, the calculated values [*Γ*]^20^_589_ corresponding to the points Q1 and Q2 in Fig. 3 are [*Γ*]^20^_589,α_(Q1) = 104.66 deg ml g^−1^ dm^−1^, [*Γ*]^20^_589,β_(Q2) = 20.74 deg ml g^−1^ dm^−1^. In the literature, the commonly found values are, respectively, [*Γ*]^20^_589,α_ = 112 deg ml g^−1^ dm^−1^, [*Γ*]^20^_589,β_ = 18.7 deg ml g^−1^ dm^−1^. Clearly, there are some reservations in the calculated absolute values, but they may stand for a good approximation placing the results into the frame of what to expect. In any case, the extracted spectral dependence *g*_11,α,β_(*λ*) may helpfully stand as the experimental counterpart to the quantities proportional to electric dipole–electric dipole and electric dipole–electric quadrupole optical activity tensors used in theoretical simulations.^30^

#### 3.2.2 Temporal dependence

Having established the spectral response throughout the mutarotation, we now focus on modeling the kinetics. Fig. 3d provides a graphical representation of the *C*_α_, *C*_β_ time evolution, as the mutarotation conversion between the d-glucose anomers proceeds. The rate constants *τ*, *τ*_⇒_, *τ*_⇐_ characterize the speed of this conversion. The *τ* is a directly fitted parameter of model (11), in which it characterizes the exponential decay (6) of the observed [*Γ*]^20^_*λ*_(*t*) spectra (Fig. 3b). The resulting fitted values for all rate constants are as follows: *τ* = 2.332 × 10^−4^ s^−1^, *τ*_⇒_ = 8.395 × 10^−5^ s^−1^, *τ*_⇐_ = 1.492 × 10^−4^ s^−1^. The fitted value of the equilibrium constant *K* = 1.777 corresponds well with the value *K* = 1.8 reported in ref. 45. Table 2 summarizes and compares our findings with some previously published values with reasonably good agreement. Ref. 42 and 29 employ polarimetry-based techniques, whereas ref. 43 and 44 are based on calorimetry and gas–liquid chromatography. As it is not possible to directly compare the latter two methods with our approach, we do not include them in our further discussion.

**Table 2:** α ↔ β d-glucose mutarotation rate constants (TW: this work)

(10^−4^ s^−1^)	TW, 20 °C	25 °C^42^	22 °C^29^	20 °C^43^	30 °C^44^
*τ*	2.332	2.473	0.767	1.867	3.517
*τ*_⇒_	0.840	0.903	0.276	—	—
*τ*_⇐_	1.492	1.570	0.491	—	—

The deviations from the tabulated values could come from several causes. Trivially, inevitable errors are due to possible inaccuracy in the concentration of the initial solution, and temperature dependence of *Γ* and [*Γ*]^20^_*λ*_. Moreover, the model does not account for the effect of the impurities in the initial powders, 96% α-d-glucose in particular. We adopt a standard approach – the composition of impurities is expected to be a racemic mixture of d-glucose, which certainly has a minor contribution to the data at the beginning of the experiment. In the view of the method demonstration and to keep the model reasonably simple, we omit this. The crucial part rests in the accuracy of *τ*, which depends on the accuracy of *Γ* (*Γ* profile forms the mutarotation exponential). Various authors report *Γ* precision of 8.3 × 10^−5^ deg cm^−1^,^29^ and 0.01 deg per 40 cm.^42^ The heterodyne polarimeter used in ref. 29 is more accurate than our ellipsometer with an accuracy of 5 × 10^−4^ deg per 5 cm. Nevertheless, at this scale, it doesn’t cause such differences among reported rate constants. Even the relatively strong deviations in *Γ*(*t*) (forming the shape of the mutarotation exponential point-by-point {*t*_1_, *t*_2_, …} and thus introducing the possible errors in a pointwise manner) cause a relatively small inaccuracy in *τ* characterizing the decay of the whole exponential formed as the *Γ*(*t*_*i*_).

Nonetheless, the coupling between spectral approach and rate equation resulting in the model (11) is what gains the upper hand over other, usually single-wavelength (typically 589 nm and 632.8 nm), polarimetric approaches. We fit the *τ* simultaneously over the hundreds of exponentials, each for a different wavelength (see Fig. 3b). In other words, we calculate *τ* from a much bigger dataset than is usually considered in other studies such as those in Table 2. Moreover, *Γ*(*λ*) follows a classical optical dispersion tendency, which results in stronger effects over the ultraviolet spectral region and weaker effects over the infrared. It benefits the signal accessibility over shorter wavelengths, particularly for rarely considered *λ* < 589 nm, providing experimental data less burdened by noise and systematic measurement errors. Monochromatic methods do not allow that at all. Thus, the robustness of our method significantly enhances the precision and sensitivity with which we calculate the rate constant *τ*.

## 4 Discussion and conclusion

In this work, we have presented an offbeat implementation of custom extended spectral Mueller matrix ellipsometry to describe the chiroptical response of simple saccharides in conjunction with evaluating corresponding mutarotation kinetics. We chose glucose, fructose, and sucrose as the method demonstration structures. We showed that the measurements resulted in a characteristic form of the Mueller matrix whose optical dispersion we have described by a physically consistent model coupled to a rate equation. We show that the spectral–temporal coupling in the model enhances the reliability, precision, and interpretation of the revealed spectra, thus enabling accurate evaluation of both the essential polarimetric quantities and the mutarotation rate. Remarkably, we extracted the gyration response of both d-glucose anomers from a single forward mutarotation reaction.

Above all, the presented method represents a universal approach applicable beyond the compounds in this study. An initial suggestion would be to adopt this method for compounds with a similar gyration response, *Γ* ′(*λ*) = 0, such as more complex saccharides. In this case, our method is ready-to-use straightaway. In principle, it would be possible to determine the individual optical response of a solution of more than one saccharide at their unknown concentrations. In such a case, however, prior knowledge of the gyration response of the individual compounds of the mixture would be necessary due to the existence of multiple combinations of fitted parameter values that would (numerically) equally describe the measured data – it is clear, that the method presented cannot replace qualitative analyses such as chromatography, but may stand as their complementary tool. In particular, it is worth extending the measurements to substances with *Γ* ′(*λ*) ≠ 0. In the spectral region given by the ellipsometer, these would typically be substances with an active chromophore and ideally exhibiting exciton coupling, enhancing the strength of the observed effect in the Mueller matrix elements *m*_14_ and *m*_41_. The spectra (12) would then be Kramers–Kronig consistent. In contrast, measurements involving substances with a hierarchical arrangement (proteins) are technically measurable, but the presented model would be inadequate to describe them. It is due to the extra contribution to overall optical activity from its secondary (tertiary) structure. Still, the effective gyration response would remain accessible, which may keep the approach more than sufficient for lots of applications. Of course, the shape of the Mueller matrix (15) would be preserved in this case as well.

We believe that despite some technical drawbacks arising from the need to navigate within the Mueller calculus framework, our approach could stand as a quick, accurate, and repeatable tool pushing the bounds of traditional polarimetry well beyond its limitations. The Mueller matrix allows simultaneous access to *Γ*(*λ*) and *Γ* ′(*λ*) separately from each other and even from other polarization effects. This paramount strength of the Mueller matrix approach has the inherent potential to open up a novel avenue for data interpretation and validation in conjunction with well-established techniques encompassing ROA, VCD, ECD, and *ab initio* calculations. For example, accurate evaluation of chemical kinetics may pave the way toward comprehensive polarimetric models for tissue characterization.

## Appendices

### A An explanatory insight into the physical aspects of the method

We are aware of the possible discouragement of the reader from applying this method in practice due to the seemingly complex-looking theory surrounding Mueller matrices. Therefore, in this section, we attempt to present the essential polarimetric principles of the present approach in an accessible and pictorial way to the reader who may not be an expert in polarimetry and ellipsometry.

#### A.1 Mueller matrix of optically active liquids

Left and right circularly polarized eigenmodes (polarization states that do not change upon interaction with given environment) propagate in a chiral liquid medium of length *d* in the form of a plane monochromatic (single-wavelength, *λ*) wave, characterized by wavenumber *k*_0_ = 2π/*λ*, with phase factor exp(−i*k*_0_*ñ*_L,R_*d*). Here, refractive indices *n*_L,R_ and absorption coefficients 
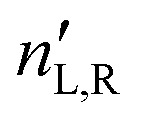
 are the respective real and imaginary parts of the complex propagation constant 
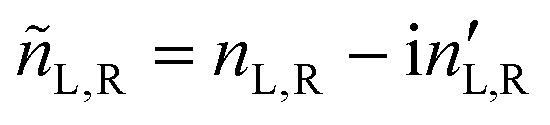
. The difference between *n*_L,R_ effectively causes an azimuthal rotation of linearly polarized light plane about angle *Γ*, while *Γ* ′ is an angular measure of the difference between 
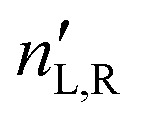
 (or the ellipticity angle) related to the eigenmodes absorption in the medium,12a
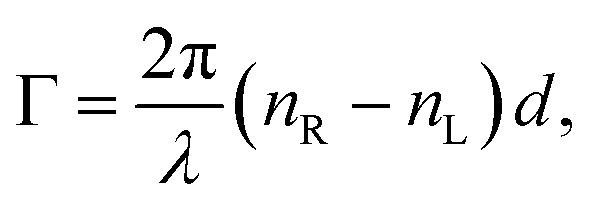
12b
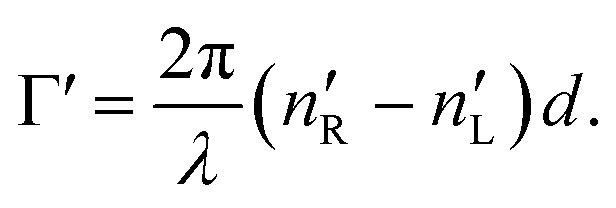


**Fig. 4:**
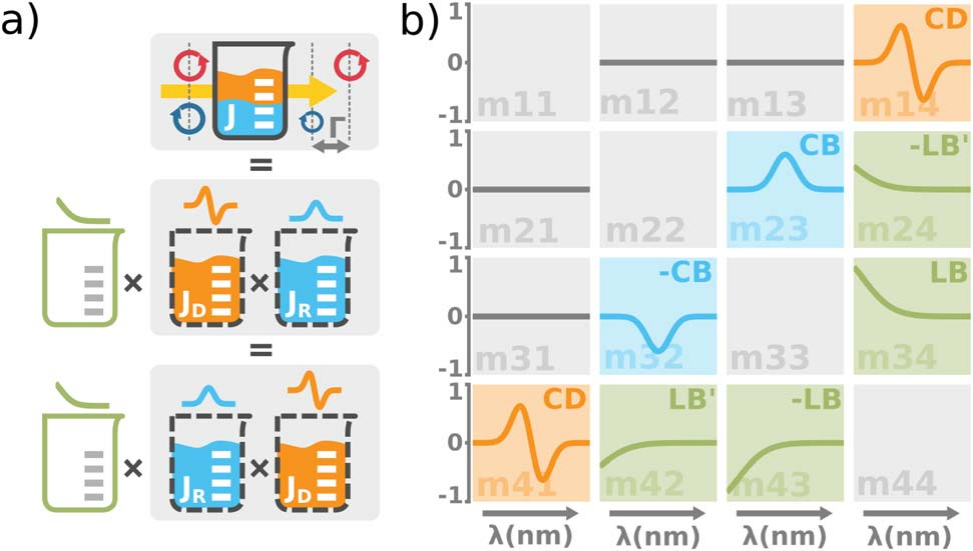
(a) The total optical response of the chiral solution can be thought of as a commuting product of the empty cuvette (linear birefringence caused by the residual stress in the glass, LB), the optically active solution (the circular birefringence causing phase retardation *Γ*, CB), and the dichroic solution (the differential absorption of left and right circularly polarized light, *Γ* ′, CD). Please take note that this is a great way for experimentalists to understand the experimental data, however, it must be used very carefully when it comes to the rigorous interpretation of the data. The observed effects must be small and measured in transmission configuration. Each effect is recorded separately within a different Mueller matrix element. (b) The graphical illustration of the Mueller matrix shows the interpretation of the relevant matrix elements (main diagonal disregarded). While the minor diagonal elements always show a chiroptical response, the green LB terms would drop to zero when using stress-free cuvettes.

The polarimetric response of the studied medium is given by transmission Jones matrix **J**. It might be hard to navigate within the Jones and Mueller matrix interpretation, if the medium possesses a complex polarimetric response. In specific cases – transmission configuration, the observed effects are small – we can think of the complex polarimetric response as a cascade of individual fundamental effects, as if the medium consisted of multiple pieces in a series, each with its own polarimetric effect. The corresponding Jones matrices multiply. For our case, this is represented in Fig. 4a – we introduce separately the effects of absorption (diattenuation, D) and dispersion (retardation, R) of circularly polarized light. The matrix **J**^LR^ (transmission Jones matrix of optically active medium expressed on the basis of circularly polarized eigenmodes) then takes the form of a product13a
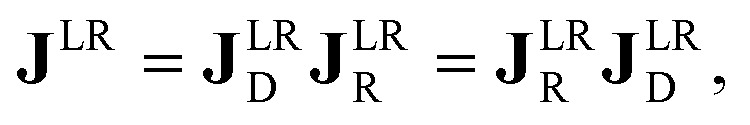
13b
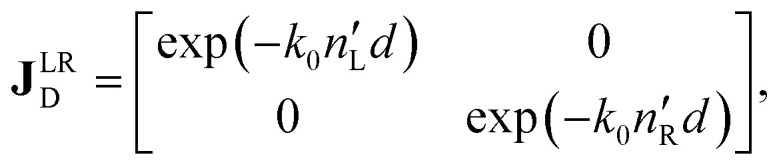
13c
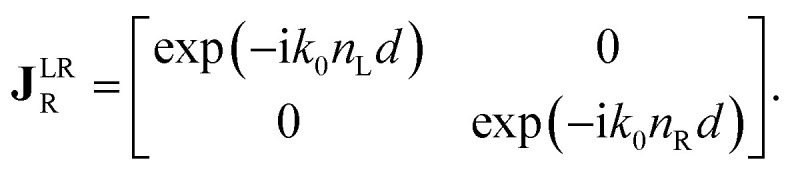
Eqn (13a) is valid, because the matrices **J**_R_, **J**_D_ commute. Now, by transforming the **J**^LR^ matrix into its Cartesian form **J**, applying some basic algebra and trigonometric identities (for detailed derivation, see ref. 46), one can obtain the following expression,14
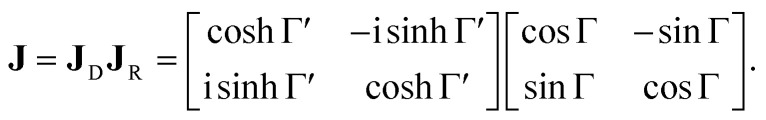


Following the well-known procedure of Jones-to-Mueller transformation 
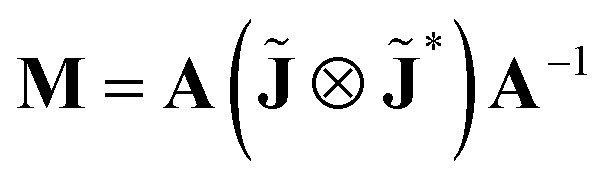
*via* matrix **A** (for details, refer to ref. 47) relating the statistical properties of light with the Stokes parameters, we obtain the Mueller matrix (normalized to *m*_11_) describing isotropic chiral media15
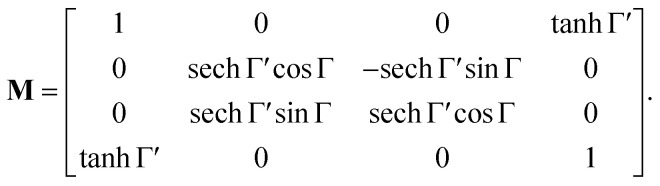


In non-absorbing spectral regions, (*Γ* ′ = 0), the matrix (14) transforms into a matrix representing standard Cartesian rotator **J** = **J**_R_, and the corresponding Mueller matrix takes the form16
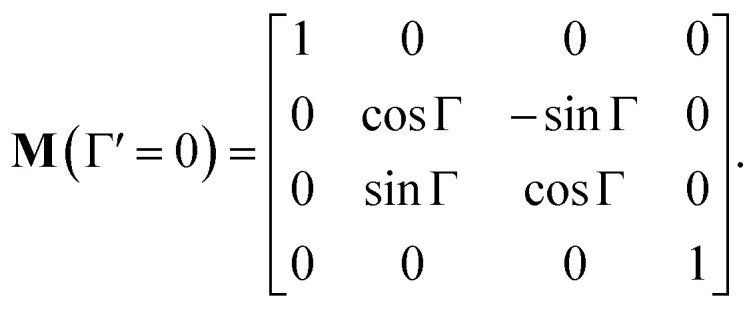
If the circular dichroism is small, it allows us to perform a Taylor expansion around the zero point, implying17
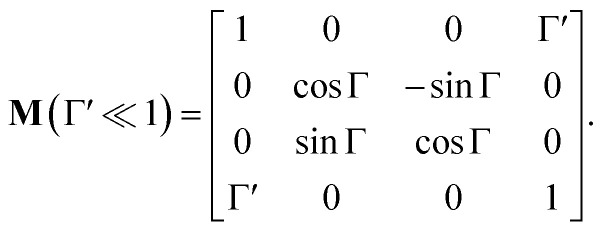


#### A.2 Chiral molecules in isotropic solution

Here we address a potential concern that may occur to some as a result of a fairly intuitive notion: since the orientation of all polarizable chiral molecules in solution is random, shouldn’t this lead to all temporal polarizations of chiral molecules averaged to zero in a given direction? If this were true, then ellipsometric measurements would be entirely inadequate for our application and would give misleading interpretation of *Γ*. To put this false doubt to rest, we offer the following explanation.

It relates the microscopic molecular quantities (in a 123 coordination system) with their macroscopic observables (in the *xyz* reference laboratory frame). To do so, we need to account for the various multipole moments within the ensemble of *N* molecules induced by the electromagnetic wave (characterized by the electric intensity **E** and magnetic flux density **B**) propagating in a solution. In the case of optically active liquid, the electric dipole *μ* and magnetic dipole **m** approximation is sufficient, and the respective moments take the form^48–50^18a
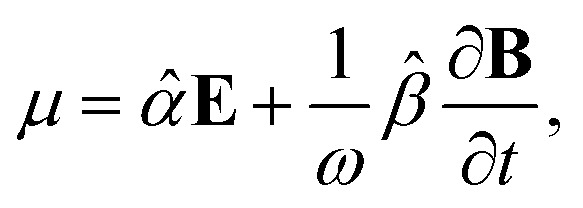
18b
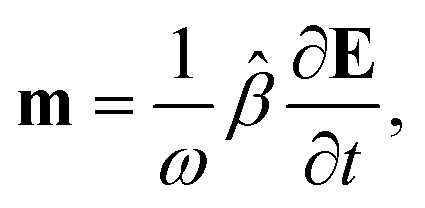
where the molecular polarizability *α̂* and optical activity tensor *β̂* are in the general form19
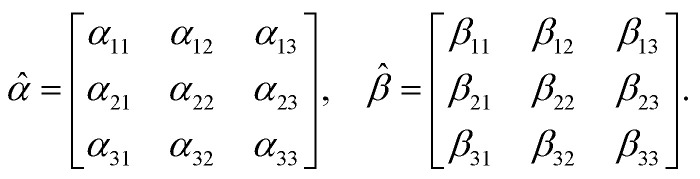
Since the orientation of each molecule in solution is random, so is the orientation of the moments, and consequently, so is the orientation of the tensors *α̂*, *β̂*.

The macroscopic polarization **P** of the chiral isotropic liquid takes the form20
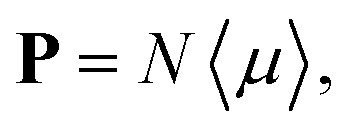
where *N* is the number of molecules and the brackets 〈…〉 denote the isotropic average (uniform over all directions). Thus, in order to calculate **P**, we need to consider the isotropic average21
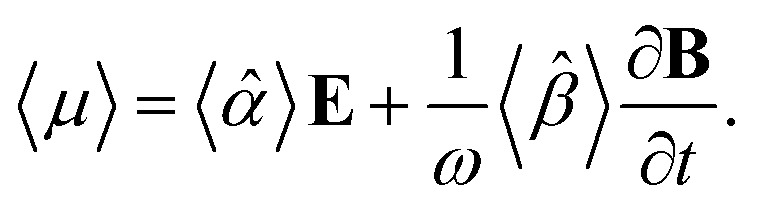
It can be shown^51,52^ that the isotropic average of any second-order tensor **û** equals22
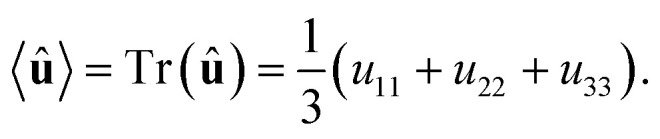


Now, to obtain the characteristic macroscopic property – macroscopic susceptibility tensor *χ̂*, we can make use of the standard definition

23 **P** = *ε*_0_*χ̂***E**,

where *ε*_0_ is the vacuum permittivity. With the help of the Maxwell equation (Faraday’s law) 
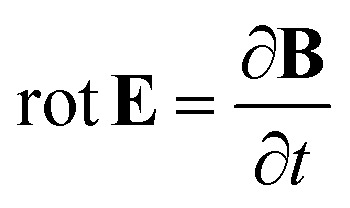
 in combination with eqn (20), we obtain24
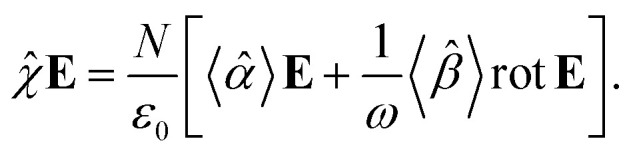
This equation can be solved for monochromatic plane waves **E** = **E**_0_e^−i**k**·**r**^, where **E**_0_ is the complex electric field amplitude, **k** is the wavevector and **r** is the position vector. Then, rot **E** ≡ ∇ × **E** = −i**k** × **E**. The cross product can be elegantly avoided by rewriting the vector **k** as follows,25
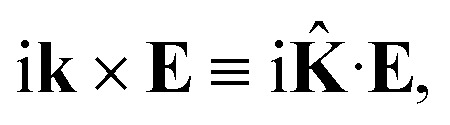
where26
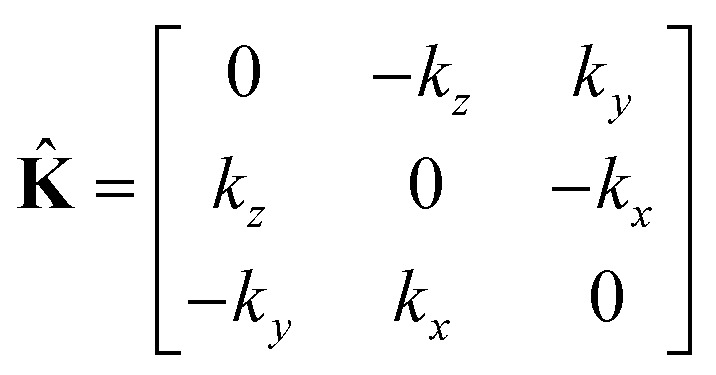
is the wave vector tensor.

The macroscopic susceptibility *χ̂*, which is a macroscopic experimental observable tied to the refractive index of the medium *via***n̂**^2^ = *ε̂* = *ε*_0_(1 + *χ̂*), then holds27
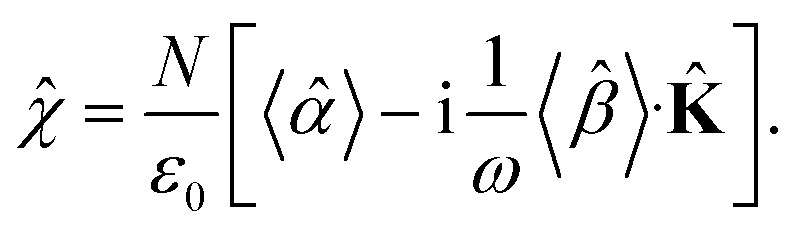
It is worth noting, that in the absence of optical activity, *i.e.* 〈*β̂*〉 = 0, the macroscopic tensor *χ̂* reduces to an isotropic diagonal tensor. In crystal optics, this is connected to the non-gyrotropic isotropic media. In chiral liquids, the contribution of the optical activity tensor *β̂*, despite being averaged to scalar quantity, arises in the off-diagonal elements of *χ̂* due to the influence of **K̂**. This is in accordance to the traditional crystal optics^53^ (classical material anisotropy) and theories of spatial dispersion^39,54^ (non-local effect of the optical activity). In our case of the light propagating in a chiral liquid along the *z* axis of the laboratory coordinate system, **k** = [001]^*T*^, therefore *χ*_13_ = *χ*_23_ = *χ*_31_ = *χ*_32_ = 0.

#### A.3 Intuitive “reading” and understanding of Mueller matrices

Strictly formally, a general Mueller matrix is a transformation matrix between two Stokes vectors, **S**^out^ = **MS**^in^, or28
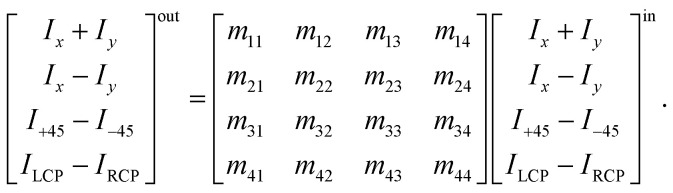
**S**^in^ defines a state of the polarized light emerging to a sample through *I*_*x*_, *I*_*y*_, *I*_±45_, and *I*_L/RCP_ denoting the wave intensities along *x*, *y*, ±45° directions, and of left and right circular polarized light, respectively. **S**^out^ bears the information about light polarization after interaction with the sample. Mueller matrix **M** carries the amount of change in different states of polarized light and thus represents a quantity that fully characterizes the sample, including depolarizations. Unfortunately, the response can be too complex in many cases and may not be easy to understand and interpret correctly.

Various numerical methods^55–57^ make the insight into the general, in our case transmission, Mueller matrix more digestible. However, these typically require a well-grounded orientation within the Jones–Mueller formalism, except for one. We have taken the liberty of stating here, without derivation, the general result of a method called differential decomposition of Mueller matrix,^58^29
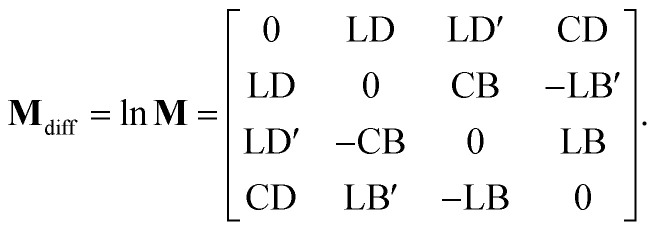
Main diagonal disregarded, the matrix elements directly correspond to dichroism (absorption) of linearly polarized modes over different sets of axes (LD, LD′), their respective birefringence (LB, LB′), and analogies for circularly polarized light (CD, CB). For our case of non-depolarizing chiral liquid, the respective elements will not directly correspond to the above quantities but will be their measures, each separately lying on the matrix secondary diagonal.

Comparing the matrix (29) with (15)–(17), the elements of interest correspond to those that work exclusively with circular polarizations. We offer an illustrative example regarding chiral solution measurements: If we use an ideal transparent cuvette without residual stress in the faces, the response of the raw experimental Mueller matrix will arise only on the secondary diagonal. Elsewise, the material stress in the cuvette (induced linear birefringence in terms of polarimetry) will give rise to signals in the elements *m*_24_, *m*_34_, *m*_42_, and *m*_43_. We illustrate this most complex case of matrix (29) in Fig. 4b. The drawn example would correspond, for example, to the response of a chiral compound with the exciton coupling causing negative bisignate CD^17^ and Kramers–Kronig related CB.

Even if we do not have a physical model, from a general acquaintance with the behavior of the polarimetric response in the Mueller matrix, it is already possible to determine the elements of our interest. Since the individual polarization effects do not mix up in most Mueller matrices, **M**_diff_ may stand for a solid clue for the experimentalist.

Note that the presented approach is rather illustrative and should be applied only to the discussed specific cases, importantly, certainly not to the reflection Mueller matrices.

## Author contributions

Daniel Vala: writing – original draft, methodology, formal analysis, investigation, data curation, visualization, conceptualization. Martin Mičica: writing – review & editing, methodology, validation, conceptualization. Daniel Cvejn: writing – review & editing, methodology. Kamil Postava: writing – review & editing, conceptualization, methodology, supervision, funding acquisition.

## Conflicts of interest

There are no conflicts to declare.

## Supplementary Material
